# IL-35 promotes CD4^+^Foxp3^+^ Tregs and inhibits atherosclerosis via maintaining CCR5-amplified Treg-suppressive mechanisms

**DOI:** 10.1172/jci.insight.152511

**Published:** 2021-10-08

**Authors:** Ying Shao, William Y. Yang, Fatma Saaoud, Charles Drummer, Yu Sun, Keman Xu, Yifan Lu, Huimin Shan, Ethan M. Shevach, Xiaohua Jiang, Hong Wang, Xiaofeng Yang

**Affiliations:** 1 Centers for Cardiovascular Research,; 2 Metabolic Disease Research & Thrombosis Research, Department of Cardiovascular Sciences, Lewis Katz School of Medicine at Temple University, Philadelphia, Pennsylvania, USA.; 3 Laboratory of Immune System Biology, Cellular Immunology Section, National Institute of Allergy and Infectious Diseases, NIH, Bethesda, Maryland, USA.; 4 Centers for Inflammation, Translational & Clinical Lung Research, Lewis Katz School of Medicine at Temple University, Philadelphia, Pennsylvania, USA.

**Keywords:** Cardiology, Inflammation, Atherosclerosis, Cellular immune response, Cytokines

## Abstract

Tregs play vital roles in suppressing atherogenesis. Pathological conditions reshape Tregs and increase Treg-weakening plasticity. It remains unclear how Tregs preserve their function and how Tregs switch into alternative phenotypes in the environment of atherosclerosis. In this study, we observed a great induction of CD4^+^Foxp3^+^ Tregs in the spleen and aorta of *ApoE^–/–^* mice, accompanied by a significant increase of plasma IL-35 levels. To determine if IL-35 devotes its role in the rise of Tregs, we generated IL-35 subunit P35–deficient (IL-35P35–deficient) mice on an *ApoE^–/–^* background and found Treg reduction in the spleen and aorta compared with *ApoE^–/–^* controls. In addition, our RNA sequencing data show the elevation of a set of chemokine receptor transcripts in the *ApoE^–/–^* Tregs, and we have validated higher CCR5 expression in *ApoE^–/–^* Tregs in the presence of IL-35 than in the absence of IL-35. Furthermore, we observed that CCR5^+^ Tregs in *ApoE^–/–^* have lower Treg-weakening AKT-mTOR signaling, higher expression of inhibitory checkpoint receptors TIGIT and PD-1, and higher expression of IL-10 compared with WT CCR5^+^ Tregs. In conclusion, IL-35 counteracts hyperlipidemia in maintaining Treg-suppressive function by increasing 3 CCR5-amplified mechanisms, including Treg migration, inhibition of Treg weakening AKT-mTOR signaling, and promotion of TIGIT and PD-1 signaling.

## Graphical abstract

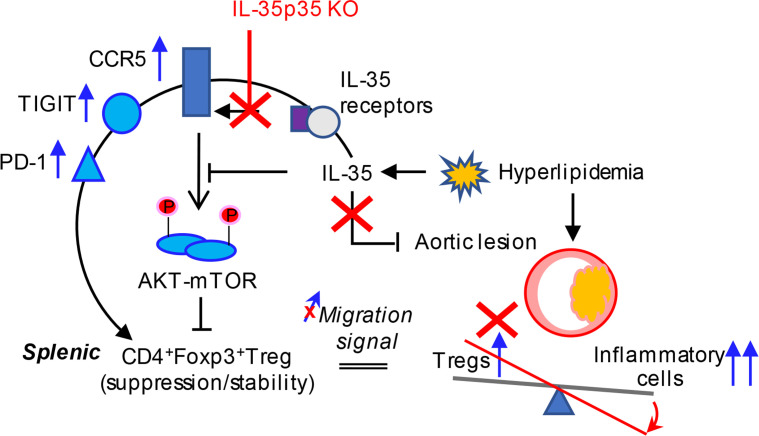

## Introduction

Immune responses mediate cardiovascular disease (CVD) risk factors such as hyperhomocysteinemia (1, 2), chronic kidney disease (3–7), hyperlipidemia (8, 9), and hyperglycemia (10, 11) to drive vascular inflammation and atherosclerosis. Tregs are specialized as the major cell type in the suppression of immunopathological reactions in the host immune system against antigens and dangers (12, 13). Following the original identification of CD25 (IL-2 receptor α chain) expression on Tregs, Forkhead Box P3 (FOXP3) has been recognized as a lineage-specifying transcription factor of Tregs (14, 15), which is critical for Tregs to maintain their immunosuppressive function. Recently, we proposed a potentially novel concept that suggests that pathological conditions/environments, via antigen epitope–dependent or –independent cellular interactions, reshape physiological Tregs into pathological Tregs that have weakened immunosuppressive functions and increased plasticity (12, 16, 17).

Significant progress has been made on the identification of Treg roles implicated in CVDs (9, 18, 19). Functional depletion of Tregs with anti-CD25 antibody results in an increase of atherosclerosis in *ApoE^–/–^* mice, suggesting that Tregs suppress atherogenesis in *ApoE^–/–^* mice (20). In addition, reports have estimated, as Treg immunosuppression targets, that 25%–38% of all leukocytes in mouse aortic and human atherosclerotic plaques are CD3^+^ T cells, with CD3^+^CD4^+^ Th cells accounting for 10% (21). However, it is still a matter of debate whether Treg subsets reach their high percentages in parallel or diminish along with the disease progression. Moreover, it also remains unclear how Tregs preserve their suppressive function and switch into alternative phenotypes in the proinflammatory environment of atherosclerosis. A very recent single-cell RNA sequencing (RNA-Seq) of aortic T cells from *ApoE^–/–^* mice fed a Western diet for 12 weeks indicated a mixed Th17/Th1/Treg transcriptome in atherosclerotic lesional T cells. Unlike apolipoprotein B-100^+^ (*ApoB^+^*) Tregs that lose a Foxp3 signal and acquire a Th1-Th17 phenotype in the atherosclerotic aorta, *ApoE^–/–^* Tregs maintain Foxp3 expression in the aorta but lose Foxp3 expression in the spleen after an adoptive transfer into 24-week-old *ApoE^–/–^* recipients after 6 weeks (22). This finding demonstrates that the hyperlipidemia environment reprograms the organ homing of functional Tregs.

IL-35 is an inhibitory cytokine that contributes to Treg function. Since Dario A. Vignali’s (University of Pittsburgh, Pittsburgh, Pennsylvania, USA) team identified IL-35 as a potentially novel heterodimeric cytokine composed of IL-35P35 and EBI3 subunits that may be specifically generated by Tregs and is required for maximal suppressive activity in 2007 (23), important progress has been made. It has been recognized that IL-35 is a responsive cytokine and is not only secreted by Treg and regulatory B cells (Bregs) (24), but to a lesser extent, is also secreted by endothelial cells (ECs), smooth muscles cells, and monocytes when prompted by proinflammatory stimuli (25, 26). IL-35 signaling is unconventional because it has multiple forms of receptors and presents cell specificity, as we reviewed in ref. 27. It can bind not only to heterodimeric receptor CD130–IL-12Rβ2, but also 2 homodimers: (a) CD130-CD130 and (b) IL-12Rβ2–IL-12Rβ2, which shows that maximal antiinflammatory function requires the heterodimeric receptor in Tregs (27). We recently reported that, via a IL-12Rβ2–dependent pathway, IL-35 delays hindlimb ischemia-induced angiogenesis but spares later regenerative angiogenesis, indicating that IL-35 also play roles in a pathological process/phase–specific manner (25, 28–33). Functionally, in addition to expanding Tregs and Bregs and modulating the imbalance between Tregs and Th cells, IL-35 is potent in inhibiting cardiovascular inflammation via its significant role in suppressing EC activation (30, 32). However, the contribution of IL-35 by regulating Treg property in atherosclerosis is unknown.

Thus, despite significant progress, several important knowledge gaps remained: (a) how hyperlipidemic atherosclerosis reshapes Treg compartmentalization; (b) how antiinflammatory cytokine IL-35 responds to hyperlipidemic environment and regulates the compartmentalization and trafficking of Tregs; and (c) whether these “atherosclerotic Tregs” maintain their immunosuppressive functions. In this study, we observed a significant induction of CD4^+^Foxp3^+^ Tregs in the spleen and aorta of *ApoE^–/–^* mice received 12 weeks of high-fat diet (HFD) feeding, accompanied with a significant increase of plasma IL-35 levels. However, the induction of Tregs was impaired in IL-35P35–deficient *ApoE^–/–^* mice. In addition, we observed a set of elevated chemokine receptor transcripts in the *ApoE^–/–^* splenic Treg RNA-Seq data and validated a higher CCR5^+^ expression in *ApoE^–/–^* Tregs — but not in IL-35P35*^–/–^*/*ApoE^–/–^* Tregs. Importantly, multiple inhibitory checkpoint receptors, including TIGIT and PD-1, have higher expression levels in CCR5^+^ Tregs compared with that of CCR5^–^ Tregs, which represent a suppressive potential. Together, our results provide convincing evidence that IL-35 promotes CD4^+^Foxp3^+^ Treg expansion and impedes atherosclerosis via CCR5-amplified suppressive mechanisms.

## Results

### CD4^+^Foxp3^+^ Tregs are increased in the spleen of ApoE^–/–^ but are decreased in blood.

**Figure 1:**
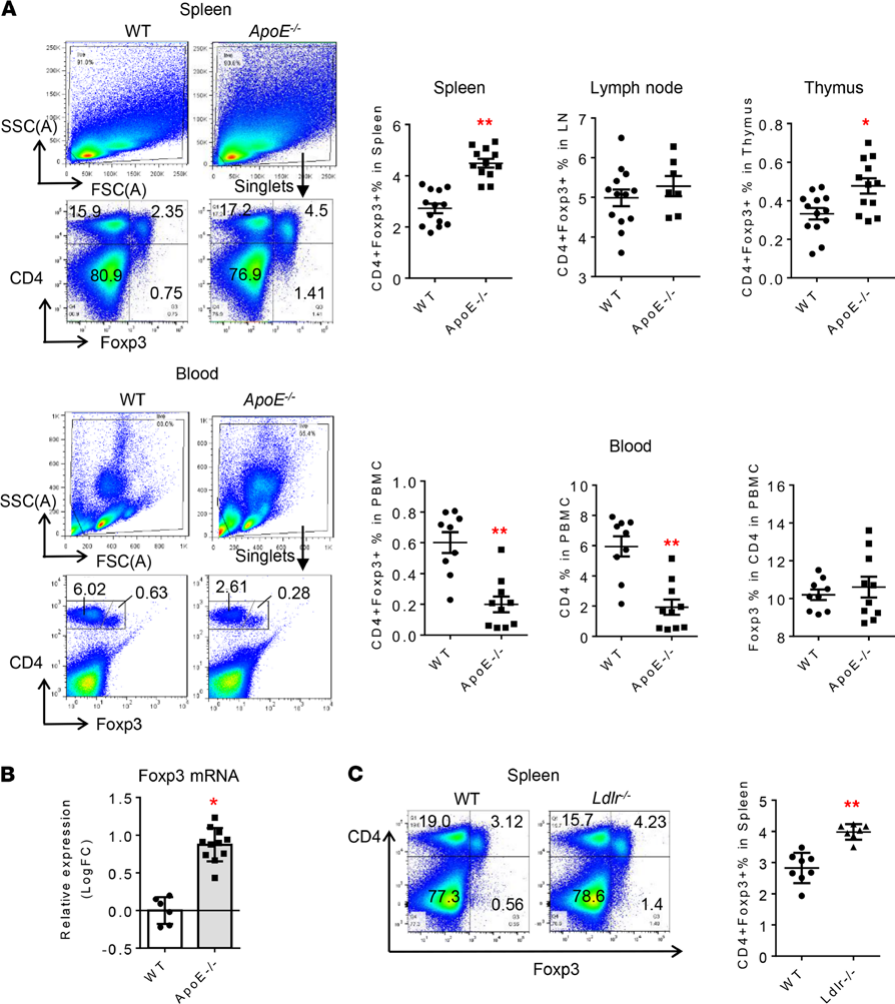
CD4^+^Foxp3^+^ Treg are significantly increased by hyperlipidemia in spleens from *ApoE^–/–^* mice and *Ldlr^–/–^* mice compared with WT controls. (**A**) Twenty-week-old mice fed with high-fat diet (HFD) for 12 weeks were investigated. The flow cytometry detections were performed for analyzing the populations of CD4^+^Foxp3^+^ Tregs in spleen, thymus, lymph nodes, and blood from *ApoE^–/–^* (*n* = 10) and WT control mice (*n* = 9). (**B**) Real-time PCRs were performed in detecting Foxp3 gene expression levels in spleen tissues in *ApoE^–/–^* mice (*n* = 11) and WT control (*n* = 6). (**C**) The flow cytometry detections were performed for analyzing the populations of Tregs in spleen from *Ldlr^–/–^* and WT control mice (*n* = 7) (*t* test; *P < 0.05, ** *P* < 0.01).

Numerous reports have shown that Tregs are hyporesponsive to stimulation with DCs (34) and are decreased in blood in atherosclerotic diseases (20, 35). Our reports show that Tregs may undergo apoptosis by decreasing the expression of anti–cell death translationally controlled tumor protein (36), and increased Treg death promotes vascular inflammation (37). These results have demonstrated that Tregs play significant roles in suppressing atherogenesis (18, 22). Our recent report indicates that Tregs have different innate immune pathways in responding to metabolic CVD risk factors, such as hyperlipidemia (6). However, the issue remains poorly characterized regarding how Tregs in peripheral lymphoid tissue maintain their homeostasis in response to hyperlipidemic conditions. To examine this hypothesis, we analyzed splenic CD4^+^Foxp3^+^ Tregs of *ApoE^–/–^* mice fed with HFD for 12 weeks and found that splenic Tregs were significantly increased in *ApoE^–/–^* mice compared with WT controls (Figure 1A and Supplemental Figure 1A; supplemental material available online with this article; https://doi.org/10.1172/jci.insight.152511DS1). In addition, thymic Tregs in *ApoE^–/–^* mice were also increased compared with WT controls; Tregs in lymph nodes were not significantly changed from that of WT controls; and Tregs in blood were significantly decreased in *ApoE^–/–^* mice compared with WT controls, presumably due to a significant reduction of CD4^+^ T cells. Moreover, *Foxp3* mRNA transcripts detected by real-time PCR (Figure 1B) in the spleen were also increased in *ApoE^–/–^* mice compared with WT controls. Since APOE was reported to inhibit inflammation in vivo, to determine whether increased Treg and Foxp3 induction result from hyperlipidemia rather than the deficiency of APOE-mediated antiinflammatory signaling (38), splenic Tregs in *Ldlr^–/–^* mice were also examined. As shown in Figure 1C, splenic Tregs in *Ldlr^–/–^* mice were significantly increased compared with WT controls. Furthermore, consistent results were observed in thymus and lymph node in *Ldlr^–/–^* mice (Supplemental Figure 1B). Of note, although we did not observe significant change of CD4^+^Foxp3^+^ Tregs in blood in *Ldlr^–/–^* mice. A similar reduction of CD4^+^ T cells was found. Taken together, these results have demonstrated that CD4^+^Foxp3^+^ Tregs were increased presumably by hyperlipidemia in *ApoE^–/–^* and *Ldlr^–/–^* spleens, but Tregs were not changed in lymph nodes and decreased in blood. Furthermore, the results show that Tregs in different tissues in *ApoE^–/–^* responded to hyperlipidemia differentially.

### IL-35 is increased in plasma of ApoE^–/–^ mice compared with WT control.

**Figure 2:**
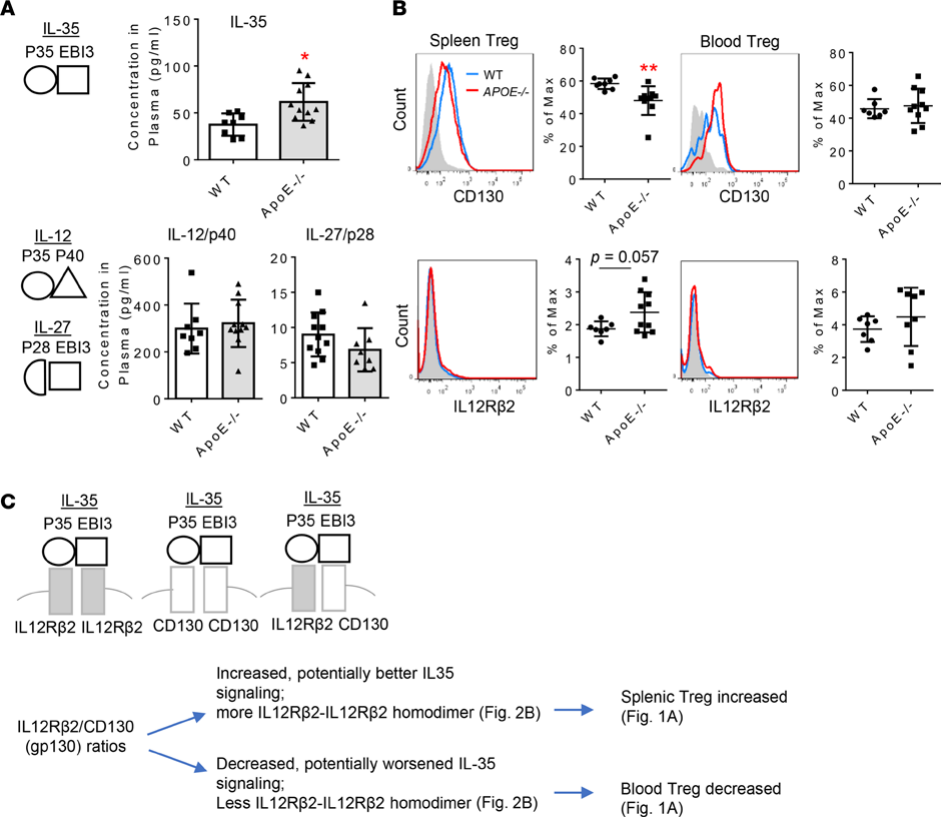
Plasma IL-35 levels are significantly increased in atherogenic *ApoE^–/–^* mice. (**A**) The ELISA was performed for detecting IL-35, IL-27/p28, and IL-12/p40 in the plasma samples from *ApoE^–/–^* mice (*n* = 11) and WT controls (*n* = 8). (**B**) Two IL-35 receptor subunits, CD130 and IL-12Rβ2, were detected in CD4^+^Foxp3^+^ Tregs in spleen and peripheral blood from *ApoE^–/–^* (red, *n* = 8) and WT mice (blue, *n* = 7). (**C**) We propose a new model based on our data that the ratios of IL-12Rβ2 over CD130 expressed on Tregs at least partially determine increase of *ApoE^–/–^* splenic Tregs and decrease of *ApoE^–/–^* blood Tregs (*t* test; *P < 0.05, ** *P* < 0.01).

We and others reported that IL-35 (30–33) is a responsive cytokine that can be induced by inflammatory diseases in several cell types, including Tregs (25, 26). Hence, we first verified whether IL-35 is induced in *ApoE^–/–^* mouse plasma as we reported for the mRNA transcripts (30) but not IL-12 and IL-27. As shown in Figure 2A, IL-35, but neither IL-12p40 (IL-12 subunit) nor IL-27p28 (IL-27 subunit), was significantly increased (61.72 ± 6.054 pg/mL) in plasma of *ApoE^–/–^* mice compared with WT controls (37.58 ± 4.201 pg/mL). We then examined IL-35 receptor expressions in Tregs. As shown in Figure 2B, IL-12Rβ2 expression slightly increased (without statistical significance) in splenic Tregs, but CD130 expression was significantly decreased in splenic Tregs of *ApoE^–/–^* mice compared with WT controls. In contrast, both IL-12Rβ2 and CD130 expression levels were slightly but not significantly increased in blood Tregs of *ApoE^–/–^* mice compared with WT controls. Of note, the expression of CD130 in both splenic and blood Tregs was more than those of IL-12Rβ2 expression. Although the future work is needed to map out the detailed composition of the IL-35 receptor in *ApoE^–/–^* splenic and blood Tregs, as shown in Figure 2C, the ratios of IL-12Rβ2 over CD130 on splenic Tregs were higher than those on blood Tregs, which correlated with the results of higher splenic Treg frequencies than blood Tregs in *ApoE^–/–^* mice. Even though the IL-35 receptor shares IL-12Rβ2 subunit with IL-12, IL-12 proinflammatory function is carried out by IL-12p40. The results of *IL-12p40^–/–^/ApoE^–/–^* mice (39) show that proatherogenic IL-12p40 is responsible for the function of IL-12 (40), and the deficiency of IL-12p40 corresponding IL-12 receptor subunit IL12Rβ1 results in immunodeficiency (41). In addition, IL-12Rβ2 plays immunosuppressive functions as IL-35 since IL-12Rβ2 deficiency leads to autoimmunity (42, 43). A Nature Medicine Paper published by another team (24), as well as our recent paper, report that *IL12rb2^–/–^* mice can be used as an *IL-35R^–/–^* model (33). Taken together, our results have demonstrated that IL-35 are increased in *ApoE^–/–^* mouse plasma, and the higher expression ratio of IL-12Rβ2 over CD130 on splenic Tregs may correlate with the higher splenic Treg frequencies in *ApoE^–/–^* mice compared with WT. These results suggest that increased IL-35 could promote splenic Tregs in *ApoE^–/–^* mice even in hyperlipidemic conditions.

### CD4^+^Foxp3^+^ Tregs are increased in the aorta of ApoE^–/–^ mice fed with HFD for 12 weeks compared with WT controls.

**Figure 3:**
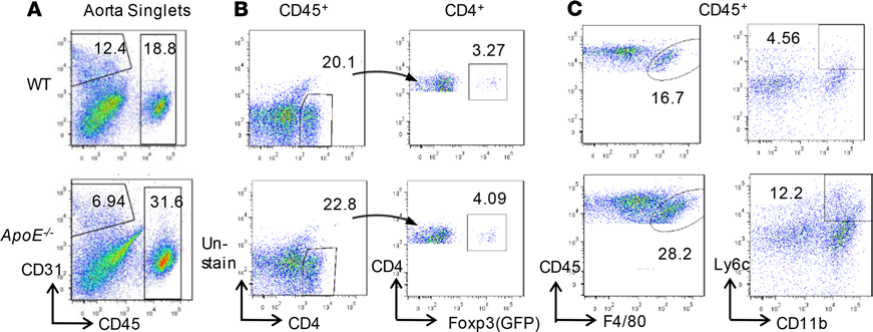
Increased CD4^+^Foxp3^+^ Treg (Foxp3 promoter-driven GFP^+^) are observed in *ApoE^–/–^* aorta along with increased leukocyte infiltrations. (**A**) Flow cytometry detection (stained for CD31 and CD45) of pooled mouse (*n* = 5/group) aortic cells. Each number indicates the percentages in the parent populations. Data were representatives from 3 separate assays. (**B**) CD4^+^ T cells and Foxp3^+^ Treg were evaluated in CD45^+^ leukocytes and CD4^+^ T cell populations, respectively. (**C**) F4/80^+^ macrophages and Ly6c^+^CD11b^+^ monocyte subpopulations were determined in aortic CD45^+^ leukocytes.

Previous reports have demonstrated that monocyte and T cell recruitment and macrophage accumulation are found in mouse aortic and human atherosclerotic plaques (21); selective depletion of Foxp3^+^ Tregs promotes atherosclerosis in mice (44). To exam whether the Treg subset reaches its high percentages in parallel to counteract its immunosuppression targets, we then hypothesized that 12 weeks of HFD can increase CD4^+^Foxp3^+^ Tregs in the aortas of *ApoE^–/–^* mice. As shown in Figure 3, A and B, CD4^+^Foxp3^+^ Tregs were increased (4.1%) in *ApoE^–/–^* aortas compared with WT controls (3.27%). In addition, hyperlipidemia also increased CD45^+^ cells (31.6%) in *ApoE^–/–^* aortas compared with WT controls (18.8%) (Figure 3A). Moreover, F4/80^+^ macrophages, CD11b^+^Ly6c^+^ monocytes, and CD4^+^ T cells were all increased in *ApoE^–/–^* aortas compared with WT controls (Figure 3C), which were well correlated with our previous reports (29, 45, 46). Due to technical difficulties in collecting enough numbers of aortic Tregs, the expression of IL-35R on aortic Tregs was not determined. These results have demonstrated that, like what we found in splenic Tregs in *ApoE^–/–^* mice, aortic Tregs are increased in *ApoE^–/–^* mice in companion with the increased frequency of aortic F4/80^+^ macrophages, CD11b^+^Ly6c^+^ monocytes, and CD4^+^ T cells.

### The deficiency of IL-35P35 significantly increases aortic atherosclerotic lesions in ApoE^–/–^ mice.

**Figure 4:**
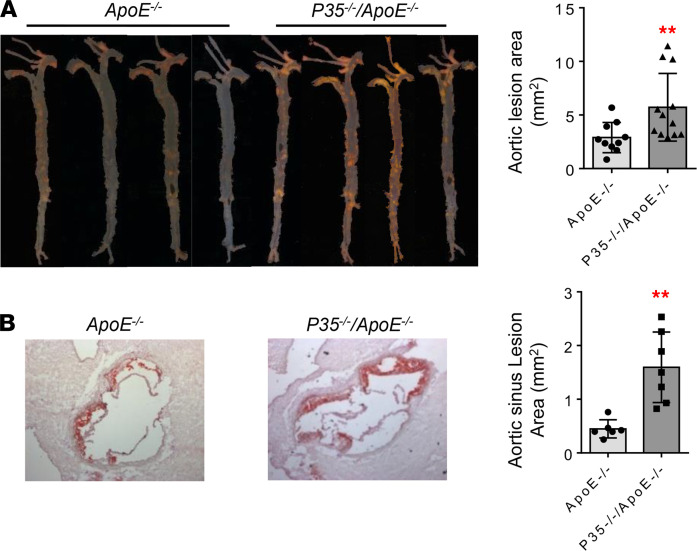
The deficiency of IL-35 subunit p35 in *ApoE^–/–^* mice significantly increases atherosclerotic lesions. (**A**) *IL-35P35^–/–^/ApoE^–/–^* mice (*n* = 10) and *ApoE^–/–^* mice (*n* = 12) were fed a HFD for 12 weeks. Representative pictures of en face Sudan IV staining of the aortas. (**B**) Histochemical Oil Red O staining of the cross section of aortic sinus (*n* = 6). The right panel shows the quantifications of atherosclerotic plaque sizes (*t* test; ** *P* < 0.01). Total original magnification, ×6.5 (**A**) and ×50 (**B**).

We and others reported that IL-35 is an antiinflammatory cytokine (27, 31, 47, 48), and *IL-35*P*35^–/–^* mice can be used as an IL-35 deficiency model since that a Nature Medicine paper reported that IL-35P35 deficiency results in defects in IL-35 and decreased Breg (24), and that IL-35P35 deficiency exacerbates cardiomyocyte apoptosis, cardiac remodeling, and mitochondrial dysfunction (49). Our IL-35 therapy model shows that IL-35 therapy inhibits atherogenesis in *ApoE^–/–^* mice (30). In contrast, a contradicting report showed that IL-35P35 deficiency ameliorates atherosclerosis (50), suggesting a need to reexamine this important issue. To consolidate our model, the deficiency of IL-35P35 in *ApoE^–/–^* resulted in a significant increase of atherosclerotic lesions detected in aortic en face staining with Sudan IV (Figure 4A) and histochemical Oil Red O staining of aortic sinus (Figure 4B). These results have demonstrated that using *IL-35P35^–/–^/ApoE^–/–^* as a loss-of-function model of IL-35, we verified that IL-35 suppresses atherogenesis in *ApoE^–/–^* mice, which are well correlated with what we reported previously using IL-35 therapy as a gain-of-function model (30).

### The deficiency of IL-35P35 significantly decreases splenic and aortic Tregs in ApoE^–/–^ mice, and this is correlated with increased CD4^+^ T cells in the aorta.

**Figure 5:**
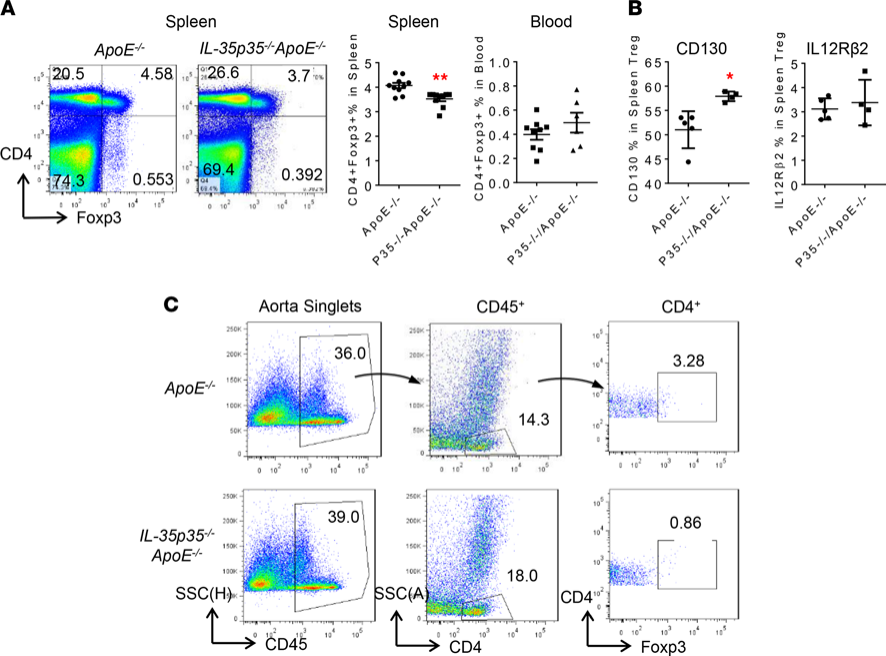
The deficiency of IL-35P35, as a loss-of-function model, decreases CD4^+^Foxp3^+^ Tregs in *ApoE*^–/–^ spleen and aorta. (**A**) Flow cytometry data found that splenic CD4^+^Foxp3^+^ Tregs are significantly decreased in *IL-35P35^–/–^/ApoE^–/–^* mice compared with *ApoE^–/–^* mice (*n* = 10). (**B**) IL-35R subunit CD130 expression was significantly increased in the *IL-35P35^–/–^/ApoE^–/–^* splenic Tregs (*n* = 4), indicated by flow cytometry results. No changes were found in the expression of IL-12Rβ2. (**C**) Representative data from a FACS assay were shown stained for CD45, CD4, and Foxp3 of pooled mouse aortic cells (*n* = 5/group). Each number indicates the percentages of the detected cell type in the parent populations (*t* test; * *P* < 0.05, ** *P* < 0.01).

To determine the mechanism underlying an increase of atherosclerosis of IL-35P35 deficiency in *ApoE^–/–^* mice, we examined Tregs in the spleen and blood. As shown in Figure 5A, splenic Tregs in *IL-35P35^–/–^/ApoE^–/–^* mice were significantly decreased compared with those in *ApoE^–/–^* control mice. Western blot also confirmed the significant reduction of FOXP3 protein levels in *IL-35P35^–/–^/ApoE^–/–^* spleen (Supplemental Figure 2A). However, blood Tregs in *IL-35P35^–/–^/ApoE^–/–^* mice were not significantly changed compared with those in *ApoE^–/–^* mice (Supplemental Figure 2B). In addition, IL-12Rβ2 expression in splenic Tregs of *IL-35P35^–/–^/ApoE^–/–^* mice were not changed, but CD130 expression in splenic Tregs was increased compared with *ApoE^–/–^* controls (Figure 5B), suggesting that IL-35 inhibits CD130 expression in Tregs. Once again, our data (Figure 2) have demonstrated that increased ratios of IL-12Rβ2 over CD130 favor an increase of Tregs. Taken together, our data have demonstrated that IL-35P35 deficiency in *ApoE^–/–^* mice decreases splenic Tregs, suggesting that IL-35 promotes splenic Tregs; IL-35 does not significantly change blood Tregs; and IL-35 promotion of splenic Tregs in *ApoE^–/–^* mice correlates well with IL-35 inhibition of atherosclerosis in *ApoE^–/–^* mice (30).

To determine the mechanism underlying increase of atherosclerosis of IL-35P35 deficiency in *ApoE^–/–^* mice, we examined Tregs in aortas. As shown in Figure 5C, the deficiency of IL-35P35 increased CD45^+^ leukocytes and CD4^+^ T cells in aortas. More importantly, CD4^+^Foxp3^+^ Tregs were significantly decreased in *IL-35P35^–/–^/ApoE^–/–^* aortas compared with *ApoE^–/–^* controls. These results have demonstrated that IL-35P35 deficiency in *ApoE^–/–^* mice not only decreases splenic Tregs, but also reduces aortic Tregs, which are well correlated with increased recruitment of CD45^+^ leukocytes and CD4^+^ T cells into aortas and increased atherosclerotic lesions in the aortas of *IL-35P35^–/–^/ApoE^–/–^* mice compared with *ApoE^–/–^* controls. Moreover, real-time PCR results (Supplemental Figure 2C) from bulk aorta revealed a lower expression of antiinflammatory cytokine *Il10* in *IL-35P35^–/–^/ApoE^–/–^* aortas compared with that in *ApoE^–/–^* mice. Unlike the unaltered expression of *Il6st* (encodes CD130), a marked upregulation of *IL12rb2* is observed in *IL-35P35^–/–^/ApoE^–/–^* aortas, suggesting that there could be a compensatory feedback response to the lack of IL-35.

### IL-35 therapy increases splenic Tregs in ApoE^–/–^ mice.

**Figure 6:**
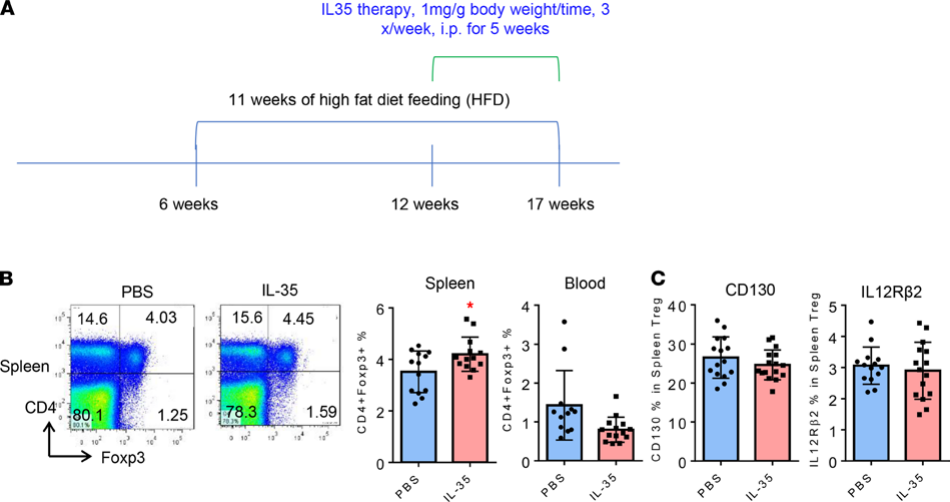
IL-35 cytokine therapy, as a gain-of-function model, increases CD4^+^Foxp3^+^ Tregs in *ApoE^–/–^* spleen. (**A**) Seventeen-week-old *ApoE^–/–^* mice with IL-35 administration for 5 weeks were investigated. (**B**) Flow cytometry found that CD4^+^Foxp3^+^ Tregs are significantly increased in spleen from *ApoE^–/–^* mice with IL-35 injection versus PBS control (*n* = 13) but are slightly decreased in blood (no statistical significance). These data correlated with our recent report, showing that IL-35 cytokine therapy significantly inhibits aortic lesions in the same experimental conditions (30). (**C**) The expressions of IL-35 receptor subunits, CD130, and IL-12Rβ2 on splenic Tregs were measured with flow cytometry, showing that IL-35 does not change the expression of IL-35 receptor subunits CD130 and IL-12Rβ2 on the splenic Treg in *ApoE^–/–^* mice (*t* test; * *P* < 0.05).

Our previous reports showed that IL-35 therapy inhibits EC activation (31), lung inflammation (32), inflammatory angiogenesis (33), and atherosclerotic lesions in *ApoE^–/–^* mice (30). Since IL-35P35 is shared between IL-35 and IL-12, to exclude the possibility of an IL-12 interference in a IL-35P35 loss-of-function model, and to directly demonstrate that IL-35 promotes Tregs, we hypothesized that IL-35 therapy increases splenic Tregs. To test this hypothesis, we adopted a reported method of cytokine therapy for atherosclerosis (51). In the last 5 weeks of the 12-week HFD feeding period, IL-35 therapy, as we reported (30), was applied to *ApoE^–/–^* mice compared with PBS controls. As shown in Figure 6, A and B, IL-35 therapy significantly increased splenic Tregs but not blood Tregs in *ApoE^–/–^* mice. Of note, additional IL-35 therapy did not change the expression of IL-12Rβ2 and CD130 in splenic Tregs (Figure 6C). Correlated with our finding that CD130 expression in splenic Tregs was significantly decreased in *ApoE^–/–^* mice compared with that of WT mice (Figure 2B) but that CD130 expression on splenic Tregs was increased in the deficiency of P35/*ApoE^–/–^* mice compared with that of *ApoE^–/–^* mice (Figure 5B), these results may suggest that exogenous IL-35 favors the induction of splenic Tregs through IL-35 receptor signaling in a CD130-tolerated mechanism. To verify this result, future experiments are required. Nevertheless, using IL-35 therapy as a gain-of-function model, our data have verified that IL-35 promotes splenic Tregs.

### Hyperlipidemia upregulates 9 top immunosuppressive classes of genes in ApoE^–/–^ Tregs compared with WT Tregs.

**Figure 7:**
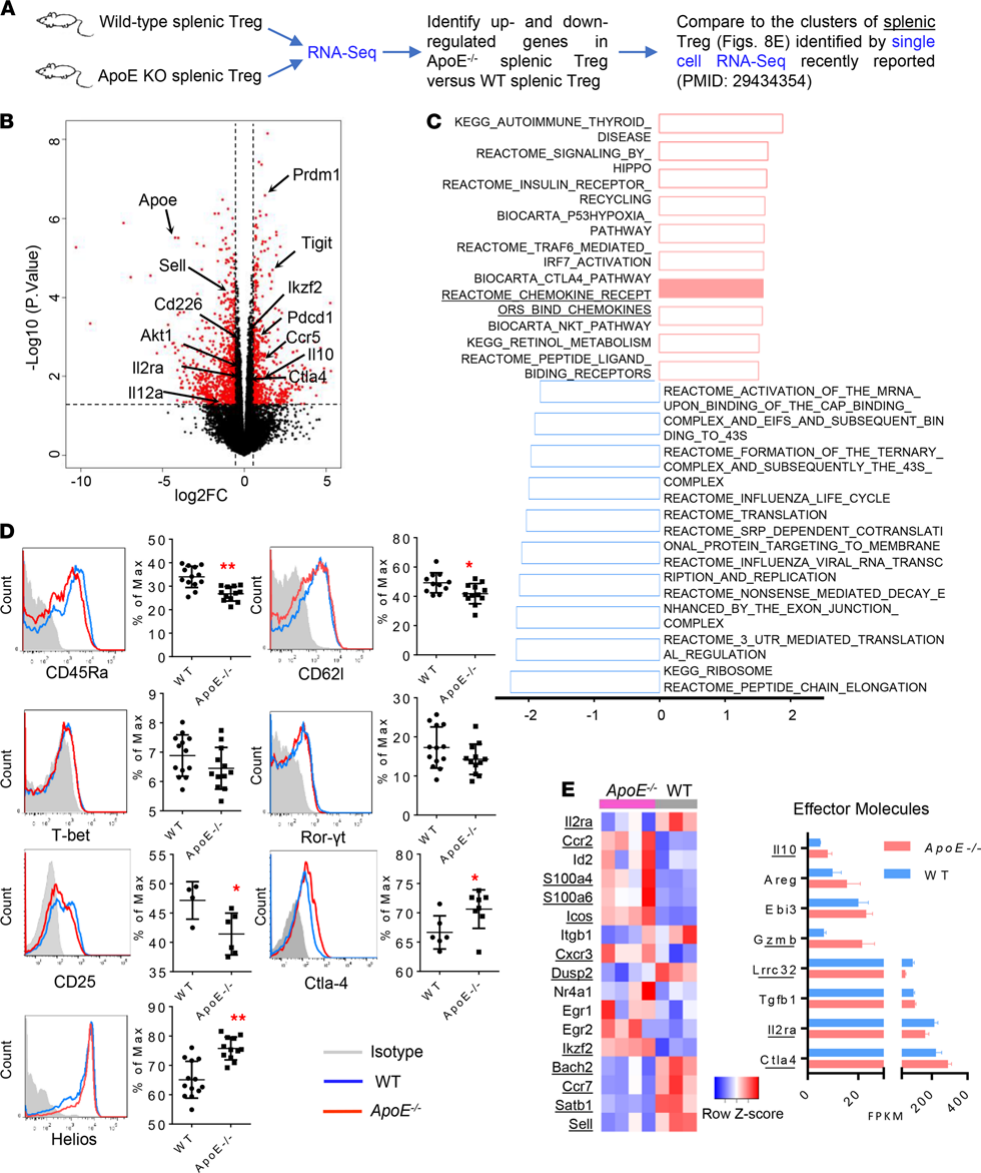
Hyperlipidemia promotes Treg transcriptomic reprogramming into more activated status with enrichment of multiple immunosuppressive classes of genes. (**A**) RNA-Seq was performed in isolated CD4^+^Foxp3^+^ Tregs from *ApoE^–/–^* (*n* = 4) and WT mice (*n* = 3). (**B**) The Volcano plot shows that hyperlipidemia significantly modulates the transcriptomes of *ApoE^–/–^* splenic Tregs versus WT controls. The differentially expressed (DE) genes with –log_10_ *P* > 1.3 (*P* < 0.05), |log_2_FC| > 0.58 (fold change, |FC| > 1.5), are labeled in red. (**C**) Gene set enrichment assay(GSEA) was performed, and top 10 classes of genes enriched in *ApoE^–/–^* (red, with positive normalized enrichment score [NES]) and WT Tregs (blue, with negative NES) were shown. (**D**) Selected marker genes such as CD45Ra, CD62l (Sell), T-bet (Tbx21), Ror-γt (*Rorc*), CD25 (*Il2ra*), Ctla-4, and Helios (Ikzf2) were verified in Tregs by flow cytometry. (**E**) Heatmap shows the mapping results to the subsets transcriptomes of the best characterized 6 clusters identified by single-cell RNA-Seq recently reported (59) showed that the most similar patterns were found in the S100a4^hi^S100a6^hi^ cluster 1 Tregs, which were with the most strongly activated phenotypes. Bar chart shows the frequencies of the expressions of Treg effector molecules from the RNA-Seq. FPKM, fragments per kilobase of transcript per million mapped reads (*t* test; * *P* < 0.05, ** *P* < 0.01).

To characterize hyperlipidemia-induced transcriptomic reprogramming in splenic Tregs of *ApoE^–/–^* mice, we performed RNA-Seq on splenic CD4^+^Foxp3^+^ Tregs of *ApoE^–/–^* mice and WT mice. As shown in Figure 7, A and B, our *ApoE^–/–^* splenic Treg RNA-Seq data showed that 1402 genes were upregulated ‚ such as *TIGIT*, *Ikzf2* (encodes Helios), *Pdcd1* (encodes PD-1), *Ctla4* (encodes CTLA-4), *Il10*, and *Prdm1* (encodes Blimp-1) — and 1539 genes were downregulated — such as *Sell* (encodes CD62l), *Il12ra* (encodes CD25), *CD226*, *Akt1* (encodes AKT), and *Il12a* (encodes IL-35P35) — in *ApoE^–/–^* splenic Tregs (*P* < 0.05) in comparison with WT controls. Using the gene set enrichment analysis (GSEA) database (http://www.gsea-msigdb.org/gsea/msigdb/index.jsp), we analyzed the top 10 classes of genes enriched for *ApoE^–/–^* splenic Tregs and WT splenic Tregs. As shown in Figure 7C, the top 10 gene classes enriched in *ApoE^–/–^* splenic Tregs included autoimmune thyroid disease, signaling by Hippo, insulin receptor recycling, P53 hypoxia pathway, TNF receptor–associated factor 6–mediated (TRAF6-mediated) IFN regulatory factor 7 (IRF-7) activation, CTLA-4 pathway, chemokine receptors bind chemokines, NKT pathway, retinol metabolism, and peptide ligand binding receptors. Of note, it has been reported that Hippo kinases Mst1 and Mst2 amplify IL-2 receptor–STAT5 (IL-2R–STAT5) signaling and stabilize Treg-suppressive functions (52). Tumor-suppressor P53-null tumors show Treg accumulation (53) and hypoxia promote Treg-suppressive function (54). Environmental insults in patients with fulminant type 1 diabetes trigger Foxp3 promoter hypermethylation, which then prevents IRF-7 binding to the Foxp3 promoter and impairs Treg development/functionality (55). NKT cells promote Tregs (56). Retinoic acid suppresses Th17 and promotes Tregs (57). Peptide binding receptors, including immune checkpoint receptors (17) CTLA-4 and T cell immunoreceptor TIGIT, promote Tregs (58). Taken together, most gene classes enriched in *ApoE^–/–^* splenic Tregs promote Treg-suppressive functions. Next, by using FACS, we confirmed the downregulation of some naive markers, such as CD45Ra and CD62l, suggesting the activation status of *ApoE^–/–^* splenic Tregs (Figure 7D). Of note, phenotypic plasticity has been reported in Tregs upon inflammatory stimulation (12, 16); however, neither T-bet nor Rorγt, the phenotypic markers for Th1 and Th17, was induced in *ApoE^–/–^* splenic Tregs. Moreover, we confirmed the induction of some immune inhibitory molecules, including CTLA-4 and Helios (*Ikzf2*), and we found a decrease trend in the deficiency of IL-35P35 (Supplemental Figure 2D). These findings suggests that *ApoE^–/–^* splenic Tregs sustain their suppressive molecules to keep the immune system in balance in the presence of IL-35.

To further identify the features of *ApoE^–/–^* splenic Tregs, we took use of the newly reported 6-cluster Tregs identified by single-cell RNA-Seq data (59). A careful comparison was made between our *ApoE^–/–^* modulated splenic Treg transcriptomic changes and the 6 clusters of splenic Treg transcriptomes (59). As shown in Figure 7E, by mapping to the subset of transcripts that best characterized the clusters, we found *ApoE^–/–^* splenic Tregs expressed higher levels of Ccr2, *S100a4*, *S100a6*, *Icos*, *Cxcr3*, and *Ikzf2* and lower levels of *Il2ra*, *Dusp2*, *Bach2*, Ccr7, *Satb1*, and *Sell*, exhibiting the most similar pattern to S100a4^hi^S100a6^hi^ cluster 1, which included the most strongly activated phenotype and most actively expressed migratory molecules. Moreover, we checked the frequencies of expression of 8 Treg effector molecules (60), as well (Figure 7E). Five of 8 effector molecules were significantly changed in *ApoE^–/–^* splenic Tregs compared with WT controls. Among them, *Clta4*, *Gzmb*, and *Il10* were increased, but *Il2ra* and *Lrrc32* (critical for tethering TGF-β to the cell surface; ref. 61) were decreased in *ApoE^–/–^* splenic Tregs. Of note, *Gmzb* (encodes granzyme B), which is involved in direct cytotoxic effects on effector T cells and APC, was greatly expanded in *ApoE^–/–^* Tregs (62, 63), suggesting that *ApoE^–/–^* Tregs may use granzyme B to lyse the target cells to suppress immune responses (13). Ultimately, a hyperlipidemic environment activated *ApoE^–/–^* splenic Tregs and reshaped their expressions of suppressive effectors.

### IL-35 promotes ApoE^–/–^ CCR5^+^ splenic Tregs, which have lower Treg-weakening AKT-mTOR signaling than that of WT CCR5^+^ Tregs.

**Figure 8:**
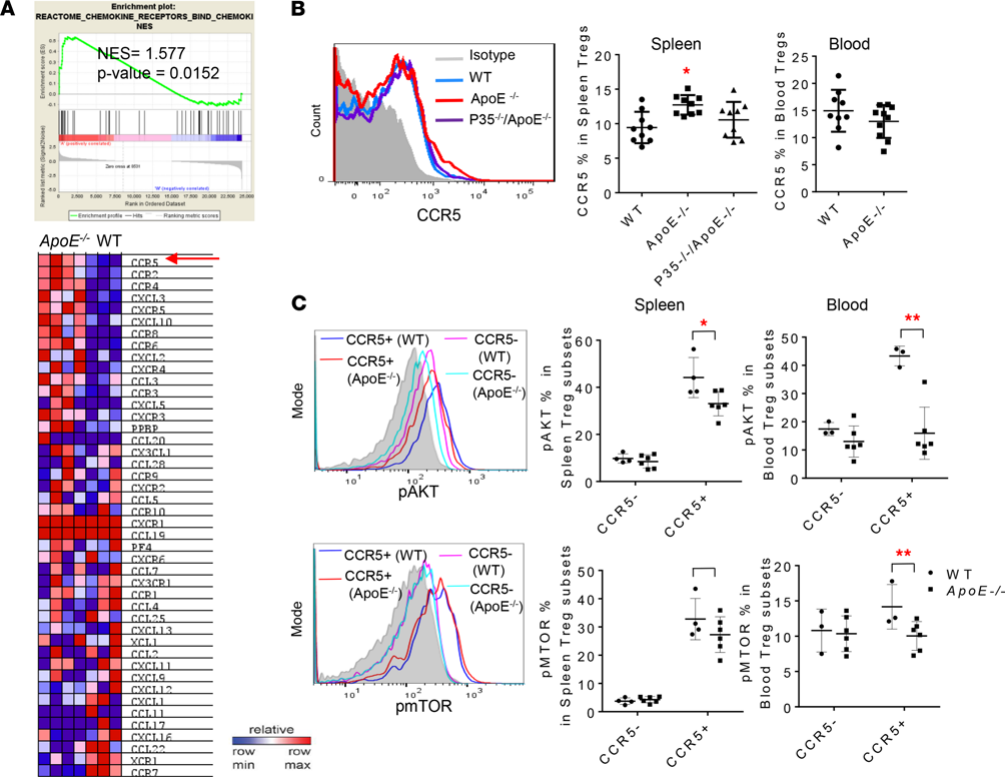
The expression of chemokine receptor CCR5 is induced in CD4^+^Foxp3^+^ Tregs from *ApoE^–/–^* mice but reduced from *IL-35P35^–/–^ ApoE^–/–^* mice. (**A**) The GSEA analysis indicates an enrichment of chemokine receptors in Tregs from *ApoE^–/–^* mice, among which the induction of *ccr5* wa*s* ranked the first. (**B**) The flow cytometry detection confirmed that Tregs from *ApoE^–/–^* mice have a higher CCR5 expression compared with WT Tregs, while the upregulation of CCR5 was limited when IL-35P35 was knocked out (*n* = 9) (1-way ANOVA; * *P* < 0.05). (**C**) Phosphorylated AKT and mTOR were detected in CCR5^+^CD4^+^Foxp3^+^ Tregs and CCR5^–^CD4^+^Foxp3^+^ Tregs, from spleen and blood by flow cytometry (*n* = 4) (*t* test; * *P* < 0.05, ** *P* < 0.01).

We previously reported that innate immune human aortic ECs activated by IL-17 and Th17 upregulate chemokines CXCL1 and CXCL2, which fulfill their roles in promoting transendothelial migration of immune cells to tissues and sites with inflammation and immune responses (64–66). Since *ApoE^–/–^* splenic Tregs matched with the activated Treg cluster genes, we hypothesized that chemokines and chemokine receptors are upregulated in *ApoE^–/–^* splenic Tregs. As shown in Figure 8A, the chemokine receptors and bind chemokines were significantly enriched in *ApoE^–/–^* splenic Tregs (normalized enrichment score [NES] = 1.577, *P* < 0.0152). In the heatmap, at least 9 chemokine receptor genes, including *Ccr5*, *Ccr2*, *Ccr4*,*Cxcr5*, *Ccr8*, *Ccr6*, *Cxcr4*, *Ccr3*, and *Cxcr3*, were significantly upregulated in *ApoE^–/–^* splenic Tregs compared with WT controls. Recent progress in the field strongly indicated that chemokine receptors play significant roles in supporting Tregs; CCR5 deficiency weakens Tregs in the spleen and brain (67). CCR2 mediates Treg recruitment to suppress inflammation in visceral adipose tissue (68). CCR4 blockade depletes Tregs (69). Treg-suppression is dependent on CXCR5 (70). CCR8 support Treg-suppression (71). CCR6 directs Treg migration to the site of inflammation (72). CXCR4 mediates Treg recruitment (73). The CCR3/CCL11 pathway increases the amount of Tregs (74). CXCR3/CXCL10 signaling mobilizes Tregs (75). Taken together, upregulations of these 9 chemokine receptors in *ApoE^–/–^* splenic Tregs increase Treg-suppressive function.

To directly examine whether chemokine receptors strengthen *ApoE^–/–^* Tregs and demonstrate the proof of principle, we focused on the top chemokine receptor CCR5 on our list (Figure 8A). To determine if IL-35 enrolls into promoting the expression of CCR5 in *ApoE^–/–^* splenic Tregs, we detected the CCR5^+^ expression in freshly prepared splenic Tregs from both *ApoE^–/–^* and *IL-35P35^–/–^/ApoE^–/–^* mice by flow cytometry. As expected, CCR5 expression on *ApoE^–/–^* splenic Tregs was increased (Figure 8B). However, the CCR5 expression on splenic Tregs from *IL-35P35^–/–^/ApoE^–/–^* mice was decreased compared with *ApoE^–/–^* controls, suggesting that IL-35 promotes CCR5 expression in *ApoE^–/–^* Tregs. Of note, CCR5 have 4 chemokine ligands — CCL3, CCL4, CCL3L1, and CCL5 (76) — among which *Ccl3* transcripts were significantly upregulated in *ApoE^–/–^* splenic Tregs (Figure 8A); this suggests the functional status of CCR5 signaling. Next, to evaluate the expression of CCR5 in Treg development, function, and stability in the activation status, we detected 2 components, protein kinase B (AKT1) (77) and mTOR (77, 78), from the PI3K-AKT/mTOR pathway, whose activation delivers a cell-intrinsic negative signal to restrain Tregs cell suppressive activity (79). As shown in Figure 8C, we found that AKT (S473) phosphorylation and mTOR (S2448) phosphorylation were lower in *ApoE^–/–^* Tregs in spleen and blood than that in WT Tregs, and specifically, the major differences were falling into the CCR5^+^ subsets. Furthermore, compared with CCR5^–^ Tregs, both WT CCR5^+^ Treg and *ApoE^–/–^* CCR5^+^ Treg populations showed higher levels of AKT and mTOR in the spleen and blood, but *ApoE^–/–^* CCR5^+^ splenic Tregs had lower levels of AKT and mTOR than WT controls. These findings suggest that CCR5 signaling may benefit AKT-mTOR phosphorylation (80) in Tregs, which promotes Tregs migration. However, AKT-mTOR signaling weakens the function and stability of Tregs (9, 81). Taking together, in hyperlipidemia status, the IL-35–promoted *ApoE^–/–^* CCR5^+^ Tregs exhibit a lower Akt/mTOR activation, suggesting that IL-35 may play a vital role in maintaining a stable Treg immunosuppressive signature in hyperlipidemia (82).

### ApoE^–/–^ CCR5^+^ Tregs have higher expression levels of immune checkpoint receptors TIGIT and PD-1 than those of ApoE^–/–^ CCR5^–^ Tregs.

**Figure 9:**
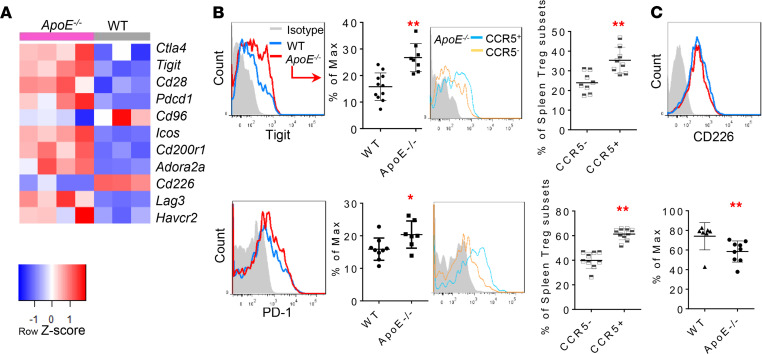
Hyperlipidemia-induced immune checkpoint receptors TIGIT and PD-1 can be further upregulated by CCR5 on Tregs. (**A**) RNA-Seq data reveal that suppressive immune checkpoint receptors rather than costimulation receptors were induced in Tregs from *ApoE^–/–^ mice*. (**B**) The flow cytometry detection confirmed that Tregs from *ApoE^–/–^* mice have higher expressions of TIGIT and PD-1 compared with WT Tregs. Moreover, both TIGIT and PD-1 were expressed higher in CCR5^+^ Tregs than CCR5^–^ Tregs (*n* = 8). (**C**) The flow cytometry detection verified that Tregs from *ApoE^–/–^* mice have lower CD226 expressions than WT Tregs, shown in graph below (*t* test; * *P* < 0.05, ** *P* < 0.01).

Immune checkpoint receptors play significant roles in regulating Tregs (17, 83–85). From the RNA-Seq data, we found 9 cosignaling receptor genes — such as *Ctla4*, *TIGIT*, *Cd28*, *Pdcd1*, *Icos*, *Cd200r1* (86), *Adora2a* (87), *Lag3*, and *Havcr2* (encodes Tim-3) (88) were upregulated in *ApoE^–/–^* splenic Tregs while *Cd96* and *Cd226* — were downregulated (Figure 9A). Except for *Cd28* and *Icos*, the rest of those upregulated cosignaling receptors belong to inhibitory immune checkpoint molecules (89). Of note, although CD28 competes with CTLA-4 for binding to shared ligands (CD80 and CD86) and transmits a stimulatory signal, it also maintains a stable pool of peripheral Tregs by both supporting their survival and promoting their self-renewal (90). In contrast, TIGIT and CD226, which both bind to CD155, counteract each other to raise or disrupt, respectively, Treg-suppression and stabilities (91, 92). Indeed, our RNA-Seq data show opposite changes of these 2 genes and indicate a high TIGIT/CD226 ratio in *ApoE^–/–^* splenic Tregs. We then examined a hypothesis that CCR5 plays important roles in promoting the expression of immune checkpoint receptors on *ApoE^–/–^* Tregs. To examine this hypothesis, we examined the expression of TIGIT and PD-1 (Pdcd1), the molecules that have been reported to mediate antiinflammatory function in Tregs (93, 94). As shown in Figure 9B, the expressions of both TIGIT and PD-1 on *ApoE^–/–^* splenic Tregs were significantly increased compared with WT controls. In addition, *ApoE^–/–^ CCR5^+^* Tregs had significantly higher levels of TIGIT and PD-1 expression levels than *ApoE^–/–^ CCR5^–^* Tregs. Since our RNA-Seq data show that CD226 expression on *ApoE^–/–^* Tregs was lower than that of WT Tregs, we also verified the results with flow cytometry (89) (Figure 9C). Taken together, our results have demonstrated that *ApoE^–/–^* Tregs upregulate 7 immune checkpoint receptor genes with Treg promoting and immune suppressive functions including *Ctla4*, *TIGIT*, *Pdcd1*, *Cd200r1*, *Adora2a*, *Lag3*, and *Havcr2* and downregulate T cell costimulation receptor *Cd226*. CCR5^+^ Tregs have higher expression levels of immune checkpoint receptors TIGIT and PD-1 than *ApoE^–/–^ CCR5^–^* Tregs.

### ApoE^–/–^ Tregs produce more IL-10, especially in CCR5^+^ subsets, than WT controls.

**Figure 10:**
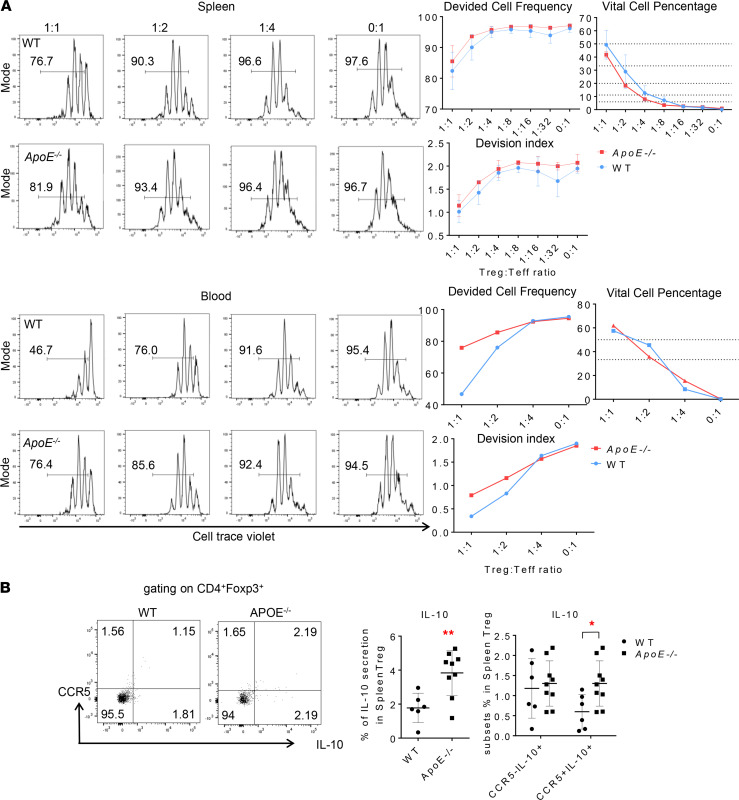
*ApoE^–/–^* Tregs from spleens show sustained suppressive functions in vitro and increased IL-10 expression. (**A**) In vitro Treg-suppression assays based on cell trace violet staining for proliferating Teffs in the presence of various ratios of WT Tregs or *ApoE^−/−^* Tregs from spleen and blood (pooled sample); the proportions of proliferating cells were shown in each panel, and data were representatives from 3 separate assays. Divided cell frequencies and percentages of Tregs were generated by flow cytometry gating, and division indexes of proliferating Teffs were calculated based on proliferation platform in the flow cytometry quantitation software flowjo.10 (https://www.flowjo.com/). (**B**) Representative flow cytometry data showed that splenic CD4^+^Foxp3^+^ Tregs from *ApoE^–/–^* mice (*n* = 9) have higher IL-10 generation than controls (*n* = 6); CCR5^+^Tregs from *ApoE^–/–^* mice have higher IL-10 generation than CCR5^–^ Treg (*t* test; * *P* < 0.05, ** *P* < 0.01).

To examine Treg-suppressive function, in vitro studies using standard Treg-suppression assays were performed as we reported (95). We found that *ApoE^–/–^* splenic Tregs have similar suppressive effects on CD4^+^Foxp3^–^ T effector cell (Teff) proliferation compared with WT Tregs (Figure 10A). To avoid testing the responsiveness of Teff to Treg from different origins, which could not happen in vivo, we used *ApoE^–/–^* Teff proliferation rates for measuring *ApoE^–/–^* Treg-suppressive function and WT Teff proliferation rates for measuring WT Treg-suppressive function (96). Teff proliferation data as Treg-suppressive function readouts were presented in both conventional gating for divided cells and division index calculated in Flowjo.10 Proliferation platform (97). Furthermore, to understand splenic Tregs peripheral organ specificity in hyperlipemia status, we performed the same setting by using Tregs and Teffs from peripheral blood. Indeed, compared with the splenic Tregs, circulating Tregs had higher suppression on Teff proliferation in both *ApoE^–/–^* and WT, whereas *ApoE^–/–^* blood Tregs had less suppressive function than WT Tregs. Of note, the marked changes were observed in the high ratios of Tregs to Teffs, which may not be physiologically relevant (97). In addition, since the in vitro coculture lasted 72 hours with no replenishment of additional IL-35, and the vital Treg ratios in the collection time were obviously lower than those in the initial mixtures, Treg stability and the cell death in vitro may affect the in vitro Treg-suppression results (97). Hence, further assays beyond simply measuring the suppression of Teff proliferation should be considered in the future.

Next, to verify our RNA-Seq data, which shows higher *Il10* expression in *ApoE^–/–^* Tregs than WT controls, as well as higher expression of *Prdm1*, a transcription factor that is critical for antiinflammatory IL-10 production and prevents proinflammatory activity in Tregs (98, 99), we detected the intracellular IL-10 expression in splenic Tregs following a 5-hour cytokine priming with flow cytometry. Consistent with the RNA-Seq results (Figure 7B), *ApoE^–/–^* splenic Tregs had a higher IL-10 secretion than WT Tregs. Of note, intracellular IL-10 expression in *ApoE^–/–^* CCR5^+^ Treg subsets was higher than that of WT CCR5^+^ Tregs, and this may contribute to higher IL-10 expression in *ApoE^–/–^* Tregs than WT Tregs, even though CCR5 signal was weaker after priming than in freshly prepared Tregs (Figure 10B). Taken together, our in vitro data have demonstrated that *ApoE^–/–^* splenic Tregs exhibit the immunosuppression functions on Teff proliferation like that of WT Tregs, but *ApoE^–/–^* Tregs produced more IL-10, especially in CCR5^+^ subsets.

## Discussion

Significant progress has been made in exploring the role of Tregs in CVDs, including increased atherosclerosis after Treg depletion (20), increased vascular inflammation when Tregs undergo cell death in disease condition (15, 35, 37, 100–102), Th17-Treg interplays (103), and Treg plasticity (16) in hyperlipidemic environments (21, 22, 104). However, an important issue remains regarding how one of the largest peripheral lymphoid organs, the spleen, maintains its Treg-suppressive functions (105–107) in hyperlipidemic conditions (6, 12). To address this issue, we made the following findings. (a) CD4^+^Foxp3^+^ Tregs are increased in *ApoE^–/–^* and *Ldlr^–/–^* spleen but are decreased in blood of *ApoE^–/–^* mice. (b) IL-35, but not IL-12p40 and IL-27p28, is increased in *ApoE^–/–^* plasma compared with WT. IL-35R subunit IL-12Rβ2 expressions are slightly increased on splenic Tregs and blood Tregs of *ApoE^–/–^* mice, while the subunit CD130 expressions are significantly decreased on splenic Tregs but are increased in blood Tregs of *ApoE^–/–^* mice compared with WT. (c) CD4^+^Foxp3^+^ Tregs are increased in *ApoE^–/–^* aorta fed a 12-week HFD compared with WT controls, while the deficiency of IL-35P35 in *ApoE^–/–^* mice decreases the Treg frequencies, along with aggravating atherosclerotic lesions and proinflammatory cell infiltration. (d) Splenic Treg expansion in *ApoE^–/–^* mice is significantly impaired by the deficiency of IL-35P35, and IL-35 therapy increases splenic Tregs. (e) *ApoE^–/–^* splenic Tregs present activated Treg properties with enriched migratory molecules, as well as increased immunosuppressive classes of genes. (f) IL-35 promotes *ApoE^–/–^ CCR5^+^* splenic Tregs by limiting the Treg-weakening AKT-mTOR signaling and promoting the expression of immune checkpoint receptors (coinhibitory receptors), such as TIGIT and PD-1, in CCR5^+^ Treg subset, along with raising IL-10 production.

**Figure 11:**
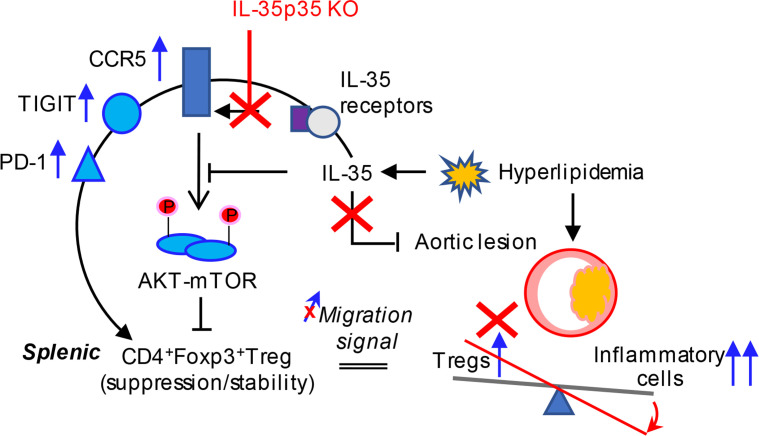
A new working model. Hyperlipidemia-induced immunosuppressive/antiinflammatory cytokine IL-35 promotes splenic Tregs and aortic Tregs in *ApoE^–/–^* mice by inducing antiinflammatory CCR5 expression on Tregs, promoting CCR5-mediated Treg migration to the aorta, inhibiting the Treg-weakening AKT-mTOR pathway, and upregulating Treg-supporting immune checkpoint receptors PD-1 and TIGIT.

Based on our results, we propose a new working model; as shown in Figure 11, IL-35 induced by hyperlipidemia counteracts atherogenesis and supports Tregs in the spleen and aorta to restrain the vascular inflammatory responses. Previously, Vignali’s team (23, 24) and others reported that IL-35 promotes Treg and Breg generation. Our previous report indicates that hyperlipidemia significantly induces mRNA transcripts of IL-35 and IL-35R subunits (27) in *ApoE^–/–^* aortas. Here, we report that hyperlipidemia induced plasma IL-35 expand the splenic CD4^+^Foxp3^+^ Tregs and support the frequencies of infiltrated Tregs in atherosclerotic aortas, which are negatively correlated with the progression of atherosclerosis (20, 37). Meanwhile, IL-35P35 deficiency (loss-of-function model) and IL-35 therapy (gain-of-function model) in vivo strongly supported this finding. In addition, we observed an increased IL-12Rβ2, which account for mediating the immunosuppressive functions (42, 43), in splenic Tregs and aortic Tregs in hyperlipidemia status; this suggests that IL-35 signaling is induced in splenic *ApoE^–/–^* Tregs and atherosclerotic aortas. As a matter of fact, we only studied the Tregs in *ApoE^–/–^* mice at a comparably early stage (12 weeks of HFD). A study on Tregs in *Lldr^–/–^* mice reported splenic Tregs increase after feeding with high-cholesterol diet for 4, 8, and 20 weeks. However, the numbers of circulating and lesion Tregs are peaked at 4 weeks and decreased significantly at 8 and 20 weeks (108). Regarding the differences between 2 atherosclerotic models (109), future work is required in order to examine the dynamic changes of IL-35 and Tregs with extended HFD feeding time.

To further determine the mechanisms underlying IL-35 promotion of *ApoE^–/–^* splenic Tregs and IL-35 maintenance of the suppressive capability of *ApoE^–/–^* splenic Tregs, we performed RNA-Seq analysis on *ApoE^–/–^* Tregs versus WT Tregs. Our RNA-Seq data show *ApoE^–/–^* splenic Tregs lose multiple naive markers and acquire activated phenotype; however, 9 of the top 10 classes of genes enriched in *ApoE^–/–^* Tregs are immune suppressive classes. We then studied the most noticeable migratory molecule CCR5 in details, which is specifically needed for CD4^+^ T cell homing to the atherosclerotic plaques (81). Several papers (9, 81) reported the plasticity of CCR5^+^ Tregs in the development of atherosclerosis, and one paper demonstrated that atherosclerosis drove the accumulation of an intermediate Th1-like IFNγ^+^CCR5^+^ Treg subset (Th1/Treg) within the aorta and peripheral lymph organs in 40 weeks of *ApoE^–/–^* mice (fed with chow diet) (9). Another paper revealed that CCR5^+^ effector CD4^+^ T cells that were exclusively found in the aorta and paraaortic lymph nodes in *ApoE^–/–^* mice with mature atherosclerotic lesions (5 months of HFD) express both T-bet and Foxp3^+^ (81). Indeed, our data reveal that, compared with CCR5^–^ Tregs, CCR5^+^ Tregs have a higher level of AKT (S473) and mTOR (S2448) phosphorylation, which have been demonstrated to be necessary for homeostatic maintenance of the Treg pools at a basal level but destabilize Tregs during the functional (suppressor) phase with excessive signaling (79, 82, 110). Specifically, PI3K/AKT activation induces the transient acquisition of Th1-like phenotypes in Tregs while maintaining the expression of Foxp3, and it inhibits Treg-suppressive capacity (111). Nevertheless, we observed that CCR5^+^ Tregs in *ApoE^–/–^* mice exhibit less AKT/mTOR activation than WT CCR5^+^ Tregs in both spleen and blood, suggesting the high levels of IL-35 in *ApoE^–/–^* mice may support CCR5^+^ Tregs to maintain their Treg-suppressive functions and stability via limiting Treg-weakening AKT/mTOR signaling.

The in vitro suppression assays indicate that *ApoE^–/–^*splenic Tregs present immune suppression on T conventional cell proliferation like that of WT controls, although splenic Tregs have lower suppression than circulating Tregs in both *ApoE^–/–^* mice and WT mice. Learning from the reprogramming of Treg effector molecules from the transcriptome analysis, we identified that the splenic Tregs in hyperlipidemia tend to exert their functions through secreting IL-10, upregulating immune inhibitory checkpoint receptors to abrogate effector T cell functions, and executing cytotoxic effects directly via granzyme B. Clearly, we have verified — with flow cytometry — the upregulation of CTLA-4, TIGIT, and PD-1 in splenic Tregs and observed a higher IL-10 generation than WT Tregs. Strikingly, all these immunosuppressive effector molecules are higher in *ApoE^–/–^*CCR5^+^ Treg subsets than that in CCR5^–^ Tregs subsets. Therefore, IL-35–promoted CCR5 amplifies immunosuppressive functions of splenic Treg in 3 aspects, including increasing CCR5-mediated Treg migration, presumably from the spleen (1000 × aortic Tregs numbers), which is a huge Treg reservoir, into the aorta; inhibiting ATK-mTOR signaling in *ApoE^–/–^* Tregs; and promoting TIGIT and PD-1 immunosuppressive functions in *ApoE^–/–^* Tregs (112). Our results have demonstrate that IL-35 promotion of Tregs is a novel mechanism for counteracting hyperlipidemia-induced weakening of Tregs, maintaining Treg-suppressive functions and Treg homeostasis. Our findings have provided insights on splenic and aortic Treg niches for performing immune suppressive function, and new therapeutic targets for CVDs, inflammation, autoimmune diseases, transplantation, and cancers.

## Methods

### Flow cytometry antibodies and reagents.

See Table 1.

### Animals.

All mice used were on a C57BL/6 background. Except in Figure 6, male mice were used in all experiments. *ApoE^–/–^* mice (stock no. 002052), *Il12a^–/–^* mice (stock no. 002692), and *Foxp3-GFP* mice (stock no. 006772) were purchased from the Jackson Laboratory. *Il12a^–/–^/ ApoE^–/–^* (*IL-35P35^–/–^/ApoE^–/–^*) and *Foxp3-GFP/ApoE^–/–^* were crossbred in our laboratory. All mice were maintained on a chow diet until 8 weeks old. For HFD feeding, they were given HFD (TD.88137, Harlan) composed of cholesterol (0.2%, w/w) and fat (21.2%, w/w), started from 8 weeks.

### Cell preparation.

To obtain single cell suspension from mouse blood, 300 μL blood was collected from mice via cardiac puncture immediately after being euthanized. Next, samples were mixed into 1 mL of PBS followed by 5 mL of ammonium-chloride-potassium (ACK) lysing buffer. After 5 minutes, 8 mL of flow cytometry (FACS) buffer was added to stop the lysis reaction. Cells were centrifugated immediately at 600*g* for 5 minutes at 4°C. The supernatant was removed, and cells were resuspended in ice-cold FACS buffer.

Freshly collected spleens were homogenized gently between the frosted ends of the slides; homogenized cells were rinsed frequently with FACS buffer. The suspended cells were transferred into a 15 mL conical tube and immediately centrifugated at 600*g* for 5 minutes at 4°C. The splenic cell pellets were lysed with 1 mL ACK for 5 minutes, followed by replenishing FACS buffer to stop the lysis reaction. Cells were centrifuged at 600*g* for 5 minutes at 4°C. The resuspended cells were passed through the cell strainer (40 μm), and the cell concentrations were adjusted to 1 × 10^6^ to 5 × 10^6^ cells/mL.

To make a single-cell suspension from the aorta, mice were perfused by cardiac puncture with 10 mL PBS containing 20 U/mL heparin. Mouse aorta (from ascending thoracic aorta to abdominal aorta) was collected and washed twice with PBS to remove blood cells in the vessel. Mouse aortas were microdissected into 5 mm length and digested in 2 mL PBS containing 20 mM HEPES/5% FBS with 2 mL/aorta of 125 U/mL collagenase type XI (MilliporeSigma, 9001-12-1), 60 U/mL hyaluronidase type I-s (MilliporeSigma, 37326-33-3), 60 U/mL DNAse1 (MilliporeSigma, 11284932001), and 450 U/mL collagenase type I (MilliporeSigma, SCR103) at 37°C for 45 minutes. The incubation mixture was shaken every 15 minutes. The digested aorta was passed through a 70 μm strainer, and the strainer was rinsed with FACS buffer. Cells were centrifuged at 600*g* for 7 minutes at 4°C**,** and the aortic cells were resuspended in ice-cold FACS buffer.

### FACS and RNA isolation for RNA-Seq.

Splenocytes from *Foxp3^gfp^* WT and *Foxp3-GFP/ApoE^–/–^* mice were stained with anti-CD4 antibody for 15 minutes at 4°C. Cell sorting experiments were performed using an Aria Cell Sorter (BD Biosciences) at Flow Cytometry Core. The CD4^+^GFP^+^ cells were sorted directly into TRIzol, and RNA was extracted using miRNeasy Mini kit (Qiagen). All original microarray data were deposited in the NCBI’s Gene Expression Omnibus database (GSE180649).

Total RNA libraries were prepared by using Pico Input SMARTer Stranded Total RNA-Seq Kit (Takara). In short, 10 ng total RNA from each sample was reverse transcribed via random priming and reverse transcriptase. Full-length cDNA was obtained with SMART (Switching Mechanism At 5′ end of RNA Template) technology. The template-switching reaction was used to keep the strand orientation of the RNA. The ribosomal cDNA was hybridized to mammalian-specific R-Probes and then cleaved by ZapR. Libraries containing Illumina adapter with TruSeq HT indexes were subsequently pooled and loaded to the Hiseq 2500. Single end reads at 75 bp with 30 million reads per sample were generated for bioinformatic analysis.

### RNA isolation and quantitative PCR (qPCR).

RNAs from flashed frozen spleen and aorta were isolated using the miRNeasy Mini Kit (Qiagen, 217004). The cDNA was synthesized using High-Capacity cDNA Reverse Transcription Kit (Applied Biosystems, 4368814), and qPCR was performed with iTaq Universal SYBR Green Supermix (Bio-Rad). Results were calculated using the ΔΔCt method relative to the reference control gene β-actin. Sequences of mouse primer pairs are included in Table 2.

### Protein extraction and Western blot analysis.

Protein extracts were collected from flash-frozen spleens. Protein concentration was determined by bicinchoninic acid (BCA) assay with BSA standards. Protein was separated on an SDS-polyacrylamide gel and transferred onto nitrocellulose membranes. Membranes were blocked with 5% BSA in Tris-buffered saline containing 0.1% Tween 20. Membranes were incubated with anti–mouse Foxp3 primary antibody (Santa Cruz Biotechnology Inc., 65988) overnight at 4°C; they were then washed extensively with TBST and incubated with the appropriate horseradish peroxidase–labeled secondary antibodies for 1 hour at room temperature. Afterward, membranes were incubated with enhanced chemiluminescence (ECL) substrate for horseradish peroxidase (Thermo Fisher Scientific, 34578), and the ECL intensity was detected by Fujifilm LAS-4000. The expression levels of proteins as indicated by the ECL intensity were measured with ImageJ software (NIH).

### ELISA.

After euthanizing mice, blood was collected through cardiac puncture and centrifuged at 3000*g* for 20 minutes at 4°C, and supernatants were carefully collected into a new 1.5 mL tube. IL-35 (Biomatik, EKU05328), IL-12p40 (R&D, M1240), and IL-27 p28/IL-30 (R&D, M2728) expression was measured following their manufacturer’s instruction.

### In vitro Treg-suppression assays.

To assess proliferation, isolated CD4^+^Foxp3^–^ (CD4^+^GFP^–^) Teffs were stained with 5 μM CellTrace Violet (Invitrogen, C34557) for 20 minutes at 37°C. In total, 2.5 × 10^4^ of CD4^+^Foxp3^+^ (CD4^+^GFP^+^) Tregs were isolated suspended in 50 μL of RPMI 1640 complete medium (Thermo Fisher Scientific, 11875093), supplemented with 10% FBS (Hyclone, SH30071.03), 50 μM 2-ME (Thermo Fisher Scientific, 2093370), 10 mM HEPES (Thermo Fisher Scientific, 15630080), 1 mM sodium pyruvate (Thermo Fisher Scientific, 11360070), 100 U/mL penicillin/streptomycin (Thermo Fisher Scientific, 15140122), and β-mercaptoethanol (MilliporeSigma, M6250) and mixed thoroughly with 50 μL of medium into round-bottom 96-well plates to generate a 2-fold dilution. The cell solution was repeatedly mixed and titrated into successive 7 wells, 50 μL at a time, leaving well 7 with no Tregs to determine maximum proliferation of Teffs. Stained Teffs were quenched with prewarmed media for 5 minutes at 37°C, and 1.25 × 10^4^ cells in 100 μL were plated in all wells. The 2 μL mouse T activator CD3/CD28 beads (Thermo Fisher Scientific, 11453D) were added to obtain a bead/cell ratio of 1:1. Each well was replenished to 200 μL media and then incubated at 37°C, 5% CO_2_, for 72 hours.

After harvesting cultures, CellTrace Violet dilution was assessed by flow cytometry and subsequently analyzed using FlowJo v 10 Software Proliferation Wizard Platform. Briefly, after sequentially gating on singlets, live cells, and CellTrace Violet^+^ cells, the percentage of responding (dividing) cells relative to the input were obtained using the provided software algorithm.

### Cytokine injection.

Recombinant mouse IL-35 (AdipoGen, CHI-MF-11135-C025) was administered via i.p. injection (1 μg/g body weight/time, 3 times per week) after 6 weeks of high-fat feeding and last for 5 weeks. An equivalent amount of PBS was injected into the control group.

### Statistics.

Data were expressed as the mean ± SEM. For comparisons between 2 groups, the 2-tailed Student *t* test was used for evaluation. For comparisons across multiple groups, 1-way ANOVA with Bonferroni post hoc test adjustment was used. Data shown were representatives of 2–3 independent experiments, including analysis from flow cytometry, qPCR, and Western blot.

### Study approval.

All animal experiments were performed in accordance with the *Guide for the Care and Use of Laboratory Animals* (National Academies Press, 2011) and were approved by the IACUC of Temple University Lewis Katz School of Medicine.

## Author contributions

Y Shao carried out the data gathering and data analysis, and prepared tables and figures. WYY, FS, CD, Y Sun, KX, YL, HS, EMS, XJ, and HW aided with analysis of the data. XY supervised the experimental design, data analysis, and manuscript writing. All authors read and approved the final manuscript.

## Supplementary Material

Supplemental data

## Figures and Tables

**Table 1:**
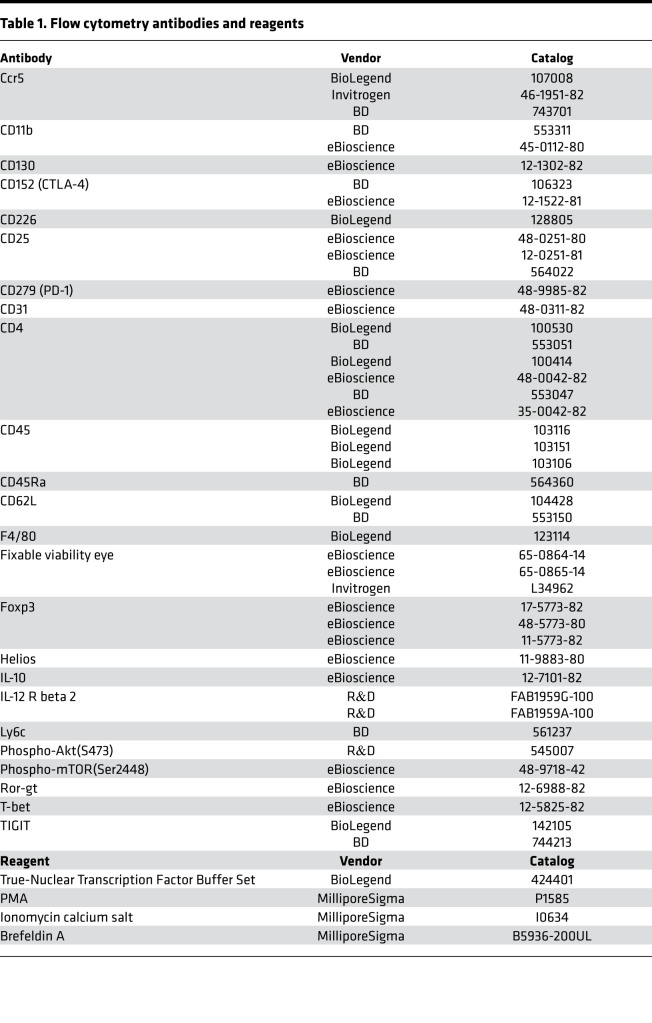
Flow cytometry antibodies and reagents

**Table 2:**
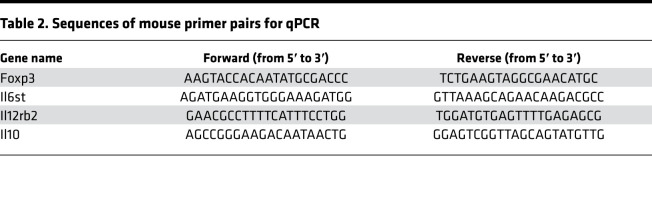
Sequences of mouse primer pairs for qPCR

## Version 1 10/08/2021

Electronic publication
