# Supplementary material for: IL-35 promotes CD4+Foxp3+ Tregs and inhibits atherosclerosis via maintaining CCR5-amplified Treg-suppressive mechanisms
Source: JCI Insight. 2021 Oct 8;6(19):e152511. doi: 10.1172/jci.insight.152511 (PMC8525592; doi:10.1172/jci.insight.152511)
Supplement: Supplemental data [file jciinsight-6-152511-s254.pdf]

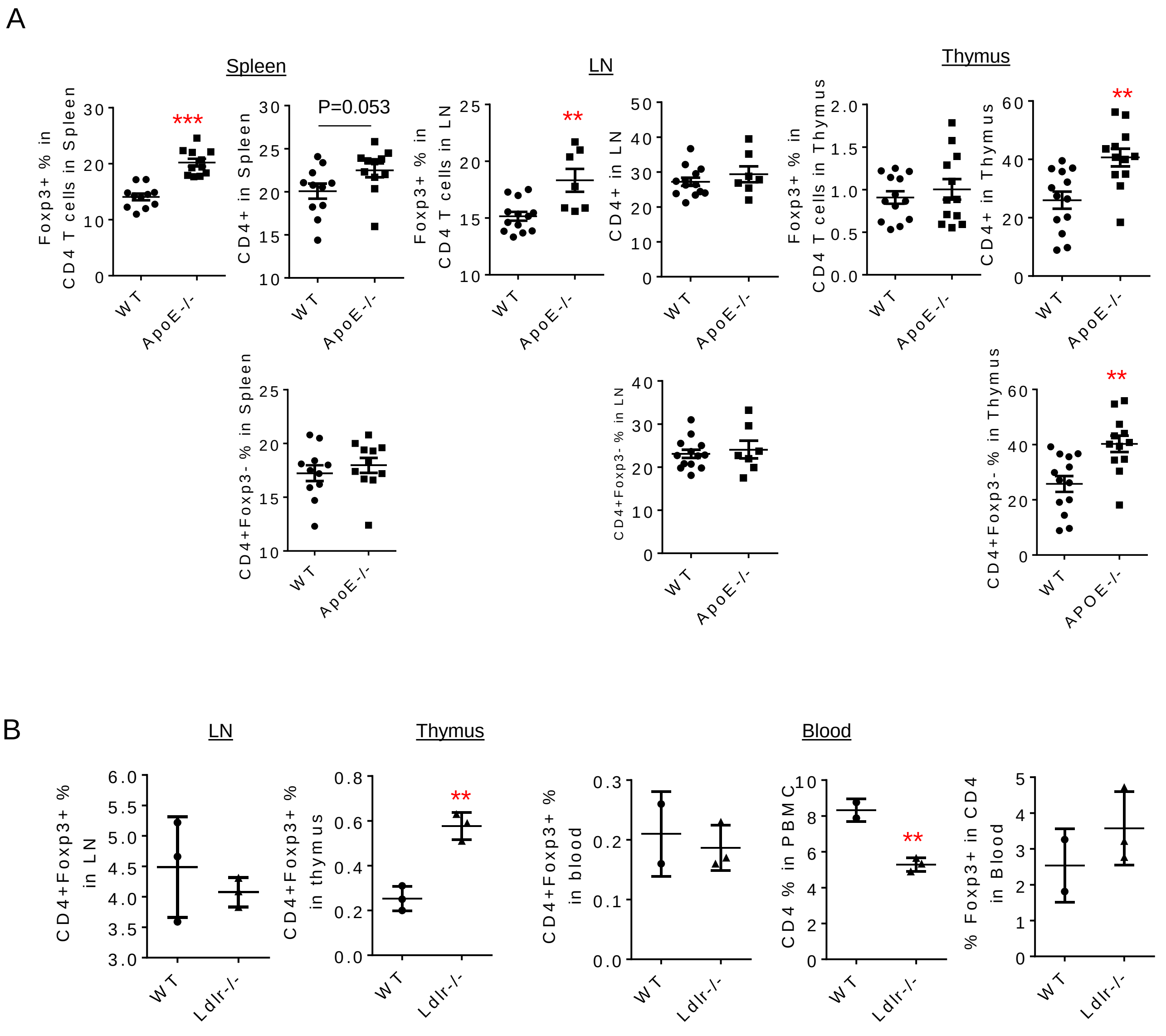

Supplementary Figure 1. The distribution of CD4$^+$Foxp3$^+$ and CD4$^+$Foxp3$^-$ T cells in spleen, lymph node and thymus from *ApoE$^{-/-}$* mice and *Ldlr$^{-/-}$ mice*. (A) The *ApoE-/-* mice of 20 weeks old fed with high fat diet (HFD) for 12 weeks were investigated. The flow cytometry detections were performed for analyzing the populations of CD4$^+$ T cells and Foxp3$^+$ Tregs, respectively (n=10). (B) The flow cytometry detections were performed for analyzing the populations of CD4$^+$ T cells and Foxp3$^+$ Tregs in The *Ldlr$^{-/-}$* mice of 20 weeks old fed with HFD for 12 weeks were investigated, respectively (n=3) (T-test, *p<0.05, ** p<0.01).

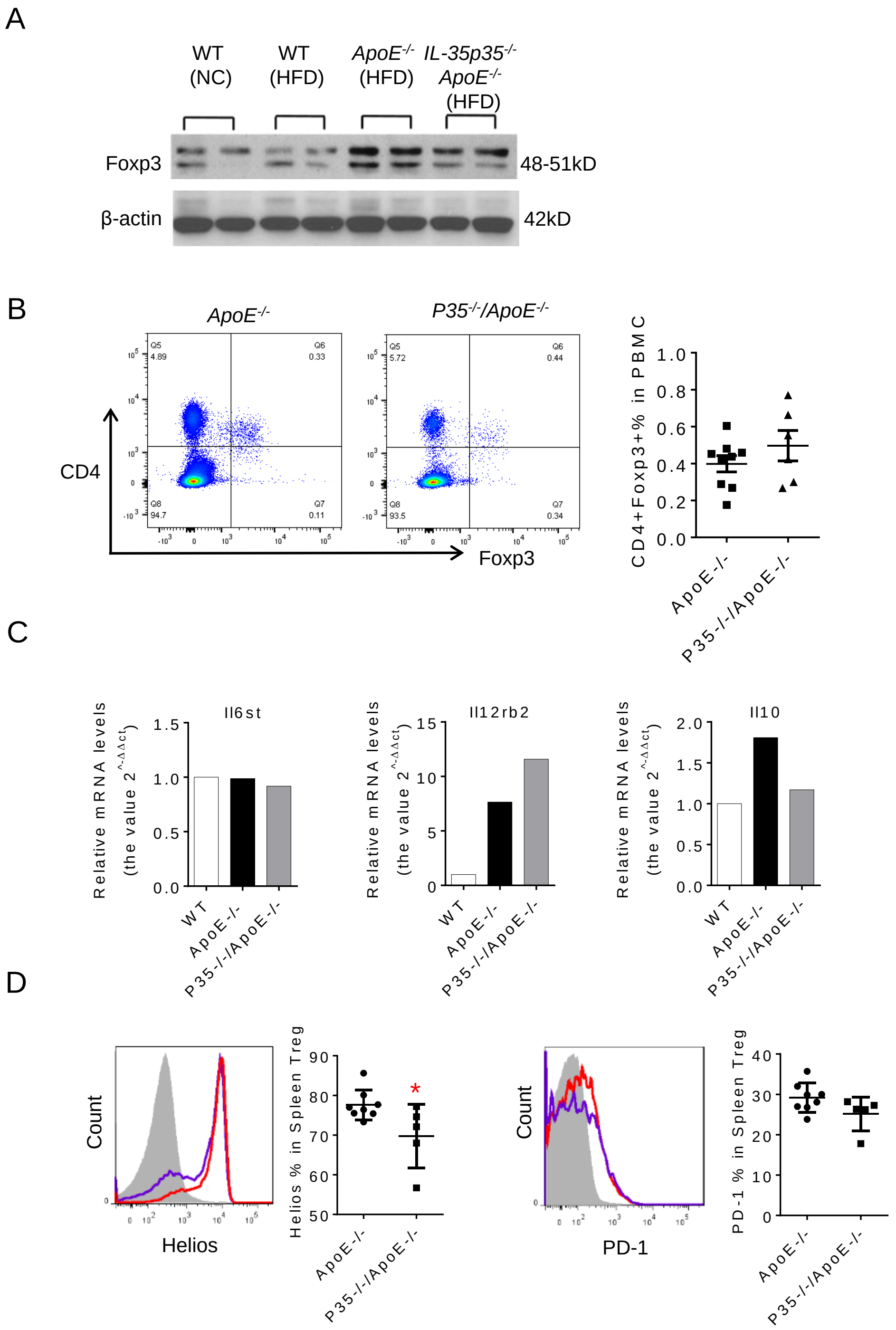

Supplementary Figure 2. Anti-inflammatory immune checkpoint receptors, such as programmed death-1 (PD-1) and Helios (Ikzf2) are found in a decrease trend in splenic Tregs in the deficiency of IL-35p35. (A) Western blots were performed in detecting Foxp3 protein levels in spleens from four groups of mice; (B) Blood Treg in ApoE$^{-/-}$ mice were not significantly changed by IL-35 p35 deficiency. The IL-35p35$^{-/-}$/ApoE$^{-/-}$ mice of 20 weeks old (n = 8) and APOE$^{-/-}$ mice (n=6) fed with high fat diet for 12 weeks were investigated. (C) Real-time PCRs were performed for detecting Il6st (encodes CD130), Il12rb2, and Il10 gene expression levels in pooled aorta samples (n = 5/group) from *ApoE*$^{-/-}$ and *IL-35p35*$^{-/-}$*/ApoE*$^{-/-}$ mice, respectively. (D) The expressions of Helios and PD-1 were examined in Treg subpopulations in freshly isolated spleens from IL-35p35$^{-/-}$/ ApoE$^{-/-}$ mice (n=5) and *ApoE*$^{-/-}$ mice, respectively, (n=8) by flow cytometry (T-test, * $p<0.05$).
